# Draft Genome Sequences of *Legionella pneumophila* JR32 and Lp01 Laboratory Strains Domesticated in Japan

**DOI:** 10.1128/genomeA.00791-16

**Published:** 2016-08-04

**Authors:** Chinatsu Maita, Mizue Matushita, Torahiko Okubo, Junji Matsuo, Masaki Miyake, Hiroki Nagai, Hiroyuki Yamaguchi

**Affiliations:** a Department of Medical Laboratory Science, Faculty of Health Sciences, Hokkaido University, Sapporo, Japan; b Department of Microbiology, School of Pharmaceutical Sciences, University of Shizuoka, Shizuoka, Japan; c Research Institute for Microbial Diseases, Osaka University, Osaka, Japan

## Abstract

We report here the draft genome sequences of two *Legionella pneumophila* variant strains (JR32 and Lp01_666) originally derived from a Philadelphia-1 clinical isolate, domesticated in Japan, with distinct susceptibility to amoebae. Detailed genomic analysis will allow us to better understand *Legionella* adaptation and survival mechanisms in host cells.

## GENOME ANNOUNCEMENT

The facultative intracellular human pathogen *Legionella pneumophila* is the causative agent of Legionnaires’ disease (1, 2). These bacteria, which inhabit the environmental reservoir *Acanthamoeba* (amoebae), enter the elderly human respiratory tract, multiply in pulmonary macrophages, and consequently cause severe pneumonia with high mortality (1, 2). Therefore, understanding the intercellular adaptation and survival mechanisms of *Legionella* is of considerable importance, prompting our interests in host-parasite relationships as well as the development of diagnostic reagents. *L. pneumophila* Philadelphia-1 strain, isolated from an outbreak in Philadelphia in 1976, is commonly used as a laboratory strain (3, 4). From the original strain, the phylogenetic diversity of the Philadelphia-1 strain has been expanded, with four major variant strains carrying distinct genomic features now available: JR32, Lp01, Lp02, and Lp03 (4). In particular, it is well known that the JR32 and Lp01 strains are successfully adapted to amoebae and human macrophages, although Lp01 exhibits 100 times less growth in amoebae (*Acanthamoeba castellanii*) than in macrophages (U-937) (5). In contrast, it has recently been observed that our lab strain, JR32, domesticated in Japan for at least 10 years, exhibits reduced growth in the same amoebae (6, 7). This suggests that strains face different selective pressures depending on individual culture conditions, leading to unique genetic changes, suggesting a useful tool for *Legionella* research. We therefore sequenced the Japanese-domesticated *L. pneumophila* strains Lp01, inserted by a green fluorescent protein (GFP) expression cassette (renamed Lp01_666), and JR32.

The draft genomes of the two *L. pneumophila* strains were obtained using an Illumina HiSeq 2000 sequencer (Illumina, San Diego, CA, USA), with sequencing runs for paired-end sequences. The bacterial DNA libraries were prepared using a TruSeq DNA sample kit (Illumina). The genomes were assembled into 44 contigs (JR32, 169 bp to 924,410 bp) and 45 contigs (Lp01_666, 169 bp to 1,132,508 bp) using *de novo* sequence assembler software (Velvet, EMBL-EBI) (8). Gene prediction, functional annotation, and comparative analysis were performed with Rapid Annotations using Subsystems Technology (RAST; http://rast.nmpdr.org/) (9). The sequencing and read assembly of the libraries were carried out by Hokkaido System Science (Sapporo, Japan).

The draft genome sequences of *L. pneumophila* JR32 and Lp01_666 strains were 3,326,989 bp (G+C content, 38.3%; coverage, 1,324-fold) and 3,345,643 bp (G+C content, 38.3%; coverage, 1,299-fold) in length, respectively. The genome sequences contained 3,153 coding sequences (JR32) or 3,175 coding sequences (Lp01_666), with 42 tRNAs and six ribosomal RNAs. A comparison with *L. pneumophila* strain Philadelphia-1 (accession no. AE017354.1) revealed that the genomes of JR32 and Lp01_666 lacked 59 and 50 genes, respectively, and contained several previously unidentified genes. This is a characteristic not previously reported for *L. pneumophila* Philadelphia-1 strain variants. More detailed analysis of the genomes will help us to understand *Legionella* adaptation and survival mechanisms in host cells, including human macrophages and amoebae.

### Nucleotide sequence accession numbers.

The draft genome sequences of *L. pneumophila* strains JR32 and Lp01_666 have been deposited in the DDBJ database under accession numbers BDFJ01000001 through BDFJ01000044 (44 entries) and BDFI01000001 through BDFI01000045 (45 entries), respectively. The versions described in this paper are the first versions.
